# Accelerating Translation of Interventions: Does It Need to Take 17 Years for Older Adults to Benefit From Science?

**DOI:** 10.1093/geroni/igaa057.1693

**Published:** 2020-12-16

**Authors:** Janet Bettger, Janet Prvu Bettger

**Affiliations:** Duke University School of Medicine, Durham, North Carolina, United States

## Abstract

Translation of evidence refers to widespread dissemination, adoption and implementation of interventions that can have a significant effect on population health. However, effective translation has been slow; significant lags and inconsistent uptake impede intended benefits for older adults. In response, interest and investments in implementation science as the study of methods to promote the adoption and integration of evidence into real-world settings have rapidly increased. By definition, the methodology applies to evidence-based practices, interventions, and policies. But the process of evidence generation can still be prolonged. This paper introduces a framework being tested at the Duke Roybal Center that integrates a model for behavioral intervention development and testing with principles of implementation science in order to accelerate translation across all phases of behavioral research. Attendees will first learn about the NIH Stage Model supported by NIA that guides researchers to identify, define, and clarify an array of activities across six stages of behavioral intervention development. These stages define components of intervention generation, pilot and then efficacy testing, effectiveness research and ultimately implementation of potent theory-driven interventions that improve health and well-being. With this foundation, the Duke framework will be presented to illustrate how concepts of several common implementation science frameworks and models can be integrated within the different stages. Interactive case studies will be used to illustrate application of this new integrated framework for evidence generation, accelerated implementation and scale-up, and pathways for translation. Integrating the Stage Model with principles from implementation science can accelerate translation.

